# Ab initio deep neural network simulations reveal that carbonic acid dissociation is dominated by minority cis-trans conformers

**DOI:** 10.1126/sciadv.adu6525

**Published:** 2025-05-07

**Authors:** Yueqi Zhao, Feifei Tian, Zhaoru Sun

**Affiliations:** School of Physical Science and Technology, ShanghaiTech University, 201210 Shanghai, China.

## Abstract

Carbonic acid (H_2_CO_3_), rather than water, serves as the primary protonating buffer regulating pH in biological systems and oceans. Its dissociation dynamics, driven by three conformers—cis-cis (CC), cis-trans (CT), and trans-trans (TT)—pose substantial experimental and theoretical challenges. Using deep potential molecular dynamics simulations with ab initio accuracy, we explored the dissociation dynamics of H_2_CO_3_ in solution on the nanosecond timescale. While the CC conformer is the most abundant, the CT conformer is the dominant proton donor. This enhanced deprotonation ability arises from the CT conformer’s involvement in more hydrogen-bonding ring structures, enabling diverse proton transfer pathways, and its greater electronic asymmetry, which increases hydrophilicity and destabilizes the hydroxyl group. Furthermore, protons dissociated from the CT conformer demonstrate a stronger preference for the homing pathway. Our findings underscore the critical role of the topology and electronic properties of the CT conformer in aqueous H_2_CO_3_ dissociation and proton transfer.

## Teaser

Exploration of the dissociation dynamics in H_2_CO_3_ solution reveals how conformer properties govern proton transfer.

## INTRODUCTION

Proton transfer is a fundamental process in various chemical, biological, and environmental systems (*1*, *2*), underpinning acid-base equilibrium (*3*, *4*), enzyme catalysis (*5*), and energy conversion (*6*). While extensive research has focused on proton transfer in water (*7*–*12*), particularly the anomalously high mobility of hydrated excess protons via Grotthuss’s “proton hopping” mechanism (*9*, *10*), recent experimental study shows that carbonic acid (H_2_CO_3_), neither water nor CO_2_, plays a more prominent role in physiological environments (*13*–*16*). H_2_CO_3_ serves as a major proton donor and is integral to the CO_2_/H_2_CO_3_/bicarbonate buffering system that regulates pH homeostasis in blood and oceans (*13*, *14*, *17*). Although extensive studies have explored carbonate under extreme pressure conditions (*18*–*21*), the dissociation of H_2_CO_3_ and the associated proton transfer mechanisms at ambient conditions remain poorly understood despite their critical biological and environmental relevance. These processes are crucial to advancing our understanding of acid-base regulation and related biological reactions.

**Fig. 1.**
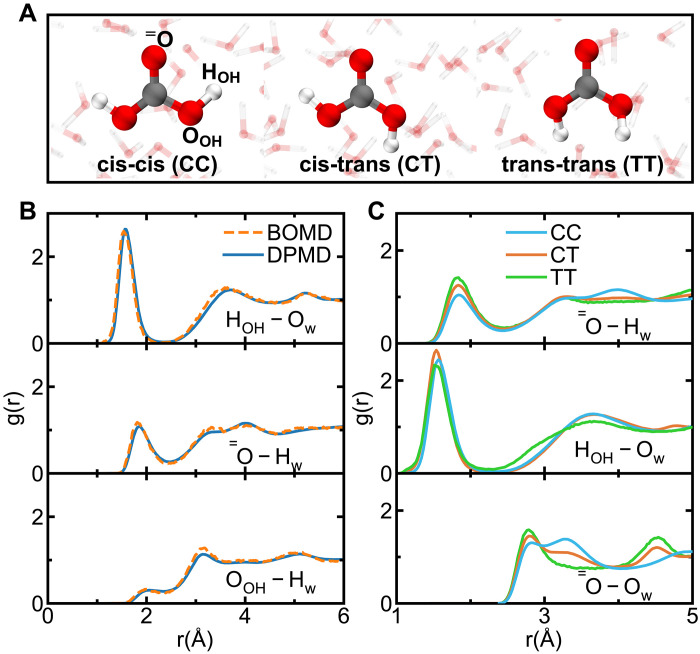
Solvation structure of H_2_CO_3_ solutions. (**A**) Molecular structures of the three conformers of H_2_CO_3_: cis-cis (CC), cis-trans (CT), and trans-trans (TT). Gray, red, and white spheres represent C, O, and H atoms, respectively. (**B**) RDFs of a CC conformer solvated by 62 water molecules obtained from 10-ns DPMD and 65-ps BOMD simulations at 330 K. O_W_ and H_W_ denote the oxygen and hydrogen atoms of the water molecules, ^=^O, O_OH_, and H_OH_ denote the carbonyl oxygen, hydroxyl oxygen, and hydroxyl hydrogen atoms of the H_2_CO_3_, respectively. (**C**) Comparison of the solvation structure for CC, CT, and TT conformers obtained from DPMD simulations.

The complexity of H_2_CO_3_ dissociation arises from the presence of three conformers (Fig. 1A): cis-cis (CC), cis-trans (CT), and trans-trans (TT), each with distinct deprotonation capabilities. While isolated H₂CO₃ molecules have inherent stability in the gas phase (*22*, *23*), extensive experimental (*24*) and theoretical (*25*, *26*) studies have consistently rank the stability of gas-phase conformers as CC > CT, with the TT conformer being unattainable in experiments because of its high relative energy (*27*, *28*). However, our understanding of carbonic acid in aqueous environments, which is essential for physiological, industrial, and geoscientific contexts, remains limited. In aqueous solutions, the intricate hydrogen-bonding (H-bonding) network markedly promotes carbonic acid dissociation (*23*, *29*), complicating the experimental differentiation of CC, CT, and TT conformers. The dynamic and cooperative nature of H-bonding network, coupled with rapid proton transfer, makes experimental characterization of specific conformers, dissociation, and proton transfer pathways in solution particularly challenging (*30*–*34*).

From a theoretical perspective, capturing the dissociation dynamics of H_2_CO_3_, especially the breaking of covalent bond, requires ab initio molecular dynamics (AIMD) simulations (*35*). However, H_2_CO_3_ dissociation occurs on nanosecond timescale (*17*), which far exceed the typical AIMD simulation window, posing challenges in accurately calculating the relative stabilities of the three conformers and in exploring the dissociation dynamics and conformational transitions of H_2_CO_3_. Traditional AIMD simulations often rely on enhanced sampling techniques to accelerate conformational transitions and explore the free energy surface (FES) (*36*, *37*), but the dependence on predefined collective variables (CVs) introduce artificial biases, leading to conflicting conclusions regarding the relative stabilities of CC and CT conformers (*38*, *39*). These biases obscure the intrinsic dynamical behavior of the system (*36*, *40*, *41*). In addition, the lack of long-range solvation effects in AIMD restricts accurate modeling of the aqueous environment around H_2_CO_3_, as water reduces dissociation free energies by strengthening H-bonds and destabilizing hydroxyl groups (*23*, *42*, *43*). Recent developments in deep potential molecular dynamics (DPMD) offer a promising alternative by combining the accuracy of first-principles calculations with the efficiency needed for long timescale simulations (*44*). DPMD maintains the integrity of the H-bonding network while capturing acid dissociation and proton transfer dynamics on nanosecond timescales (*35*), thus overcoming the limitations of traditional AIMD and enhanced sampling methods and offering deeper insight to understand the complex behavior of carbonic acid in aqueous environments.

In this work, we have trained a neural network–based deep potential (DP) model for H_2_CO_3_ in aqueous solution with ab initio accuracy and used it to investigate its conformational equilibrium, dissociation dynamics, and proton transfer. Our simulations span hundreds of nanoseconds without any bias potential, overcoming the time limitations of traditional AIMD simulations (*38*, *39*, *43*) to capture rare dissociation events, while preserving the intrinsic dynamical properties of the system. This study aims to elucidate the role of the hydrogen-bonding network in governing the dissociation and proton transfer dynamics of H_2_CO_3_ in aqueous environments. Our results show that while the CC conformer is the most stable in solution, the CT conformer exhibits higher proton-donating ability and serves as the primary proton provider in solution. This enhanced proton-donating ability of the CT conformer is attributed to two key factors: its participation in more H-bonding ring structures and an increased asymmetry in its electronic structure. In addition, we present a detailed analysis of the proton transfer pathways, revealing a stronger preference of protons dissociated from CT conformer for the homing pathway.

## RESULTS

### DP model validation

We trained the DP model using energies and atomic forces data obtained from density functional theory (DFT) calculations and Born-Oppenheimer molecular dynamics (BOMD) simulations. The training data spanned diverse configurations, including stable states (e.g., CC, CT, TT, HCO_3_^−^, and H_3_O^+^) and transition states relevant to the acid dissociation reaction pathway, to ensure comprehensive coverage of the configurational space. All training data were generated using the Strongly Constrained and Appropriately Normed (SCAN) functional (*45*), which accurately describes the structure, thermodynamic and reaction dynamics of liquid water (*46*, *47*) and aqueous systems (*48*–*50*). The DP model was validated with DFT predictions, achieving root-mean-square errors of 0.28 meV per atom for energies and 0.057 eV/Å for forces, achieving benchmark accuracy for DP models in aqueous systems (*48*, *50*–*52*) (details of the training set preparation in the Supplementary Materials).

To further validate the quality of our DP model, we conducted BOMD and DPMD simulations of the CC, CT, and TT conformers of H_2_CO_3_, each solvated by 62 water molecules, in the NVT ensemble at 330 K. We compared the radial distribution functions (RDFs) of acid-water and water-water pairs obtained from these simulations. As shown in Fig. 1B, three types of H-bonds (H_OH_–O_W_, ^=^O-H_W_, and O_OH_-H_W_) are analyzed for the solvation of the CC conformer. Additional RDFs for solutions with CC, CT, and TT conformers are shown in fig. S2. Obviously, the DP model accurately reproduced the main features of the acid-water RDFs obtained from BOMD. Minor discrepancies between DPMD and BOMD were observed, which can be attributed to the relatively large fluctuations in the RDFs predicted by the short BOMD simulation (65 ps), while the extended DPMD simulation (10 ns) enables us to obtain better statistics for the solvation structure. Furthermore, we observed that the hydroxyl oxygen atoms (O_OH_) of H_2_CO_3_ tend to form stronger H-bonds with surrounding solvent water, while the carbonyl oxygen atom (^=^O) tends to accept stronger H-bonds from the surrounding solvent water compared to the O_OH_. These trends are consistent with previous Becke-Lee-Yang-Parr (BLYP)–based Car-Parrinello molecular dynamics simulations (*38*, *53*, *54*).

The DP model effectively reveals differences in the solvation structures of the CC, CT, and TT conformers of H_2_CO_3_ in solution (Fig. 1C), offering clearer insights than BOMD simulations (see fig. S5). Previous studies have reported steric effects in the hydration shell of H_2_CO_3_ (*53*), and our unbiased DPMD simulations support these observations while providing improved convergence. As shown in Fig. 1C, the CC and TT conformers exhibit the lowest peak intensities (g_1_^max^) in the ^=^O-H_W_ and H_OH_-O_W_ RDFs, respectively. For the CC conformer, this is due to water molecules that donate H-bonds being spatially constrained between those accepting H-bonds from the H_OH_, weakening the H-bonds on that side of H_2_CO_3_. Similarly, steric hindrance in the TT conformer weakens the H-bonds formed by H_OH_, resulting in the weakest H-bonds among the three conformers. Figure S6 provides illustrative snapshots of these steric effects. Further evidence of steric influence appears in the ^=^O-O_W_ RDFs: The second peak at 3.28 Å corresponds to water molecules forming H-bonds with the hydroxyl hydrogen in the cis orientation, which impedes ^=^O’s H-bond formation with the nearest water molecule (first peak at 2.83 Å). Therefore, the CC conformer exhibits the lowest intensity for the first peak but the highest for the second, reflecting its unique solvation structure.

### Relative stability of conformers

**Fig. 2.**
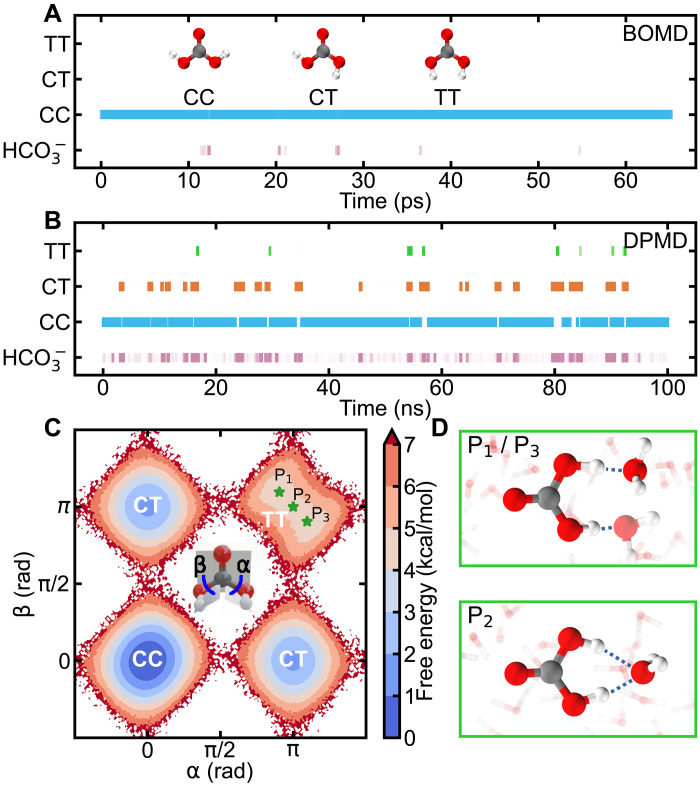
Spontaneous dissociation events and FES of H_2_CO_3_. Time evolution of H_2_CO_3_ conformers: CC (blue), CT (orange), TT (green), and HCO_3_^−^ (purple) in(**A**) BOMD simulation with 1 H_2_CO_3_ solvated by 62 water molecules in NVT ensemble at 330 K and (**B**) DPMD simulations with 1 H_2_CO_3_ solvated by 126 water molecules in NPT ensemble at 330 K and 1 bar. (**C**) FES of the three conformers of H_2_CO_3_ in aqueous solution as a function of two ^=^OC-O_OH_H dihedral angles α and β. (**D**) Two distinct solvated TT conformers: nonplanar configuration (top) and planar configuration (bottom).

The conformational transitions and dissociations of different conformers (CC, CT, and TT) are rare events and difficult to observe on the timescale of picosecond BOMD simulations (*38*, *53*, *55*). Previous studies (*55*) have shown that the CC conformer resists dissociation and conversion to other conformers unless a bias potential is imposed by metadynamics. In our 65-ps BOMD simulation starting with the CC conformer (Fig. 2A), we observed transient deprotonation events occurring at 12, 20, 27, 36, and 55 ps. In each case, the dissociated proton quickly returned to the CC conformer, as the H-bond between the proton and HCO_3_^−^ remained unbroken. Neither the CT nor the TT conformer was observed in the relatively short BOMD simulation. Additional BOMD simulations starting with CT and TT conformers similarly failed to capture transitions between CC, CT, and TT, instead showing only trajectories dominated by the selected initial conformer and HCO_3_^−^ (see fig. S7). Therefore, to gain deeper insights into the conformational transitions and dissociation dynamics of H_2_CO_3_, conducting simulations on longer timescales with DFT-level accuracy is essential.

Using DPMD simulations, we have captured abundant H_2_CO_3_ microstates and frequent conformational transitions that occur in solution on the nanosecond timescale. We performed three independent DPMD simulations starting with the CC, CT, and TT conformers solvated by 126 water molecules, respectively, each with a duration of 100 ns in the isothermal-isobaric (NPT) ensemble at 330 K and 1 bar. One typical trajectory with the CC conformer as starting state is presented in Fig. 2B, and the other two DPMD runs are shown in fig. S8. We identified multiple state changes occurring at a frequency ranging from approximately 1 ps to 10 ns. The CT and TT conformers were observed to form spontaneously, which were not captured in the BOMD simulations. We further quantified the relative abundance of carbon species based on all three DPMD trajectories by calculating their mole percent according to *x_i_* = *n_i_*/*N*, where *n_i_* is the number of the snapshots containing the *i*th species and *N* is the total number of snapshots. We found that the CC conformer dominates in aqueous solution with a mole percent of 81.3%, followed by the CT conformer (12.4%) and the TT conformer (0.3%). We also detected CO₃^2−^ with a mole percent of less than 0.01%, indicating weak second dissociation. To assess the thermal stability of conformer populations within biologically and environmentally relevant temperature ranges, we performed additional DPMD simulations at 310 and 350 K (details in the Supplementary Materials). The results reveal a slight decrease in the mole percent of CC conformers with increasing temperature, accompanied by a slight increase in the mole percent of CT and TT conformers. The CC conformer remains more abundant than the CT conformer across the investigated temperature range.

The relative stability of the three conformers is further confirmed by FES calculations, which reveal that the TT conformer in aqueous solution forms a dual H-bonding structure with a single water molecule, enhancing its stability in the aqueous phase. As shown in Fig. 2C, we presented the FES as a function of two ^=^OC-O_OH_H dihedral angles α and β of H_2_CO_3_. Both the CC and CT conformers exhibit a preference for planar configurations, with their lowest energy states detected at (α, β) = (0, 0) and (α, β) = (0, π) or (π, 0), respectively, which is consistent with previous studies (*25*–*27*, *56*). In contrast, the TT conformer exhibits multiple metastable structures, with a saddle-shaped free energy basin. Figure 2D presents two typical metastable structures for the TT conformer. One is located on the either side (P_1_/P_3_) of the saddle point and represents a nonplanar configuration, with H_OH_ forming H-bonds with two different surrounding water molecules. The other structure, located at the saddle point (P_2_), represents a planar configuration with the H_OH_ forming dual H-bonds with only one nearby water molecule. Similar dual H-bonding structures have been previously reported in H_2_CO_3_-halide complexes through infrared action (*57*) and negative ion photoelectron spectroscopy (*58*), where both hydroxyl groups form H-bonds with halide anions, markedly enhancing the stability of the TT conformer. Consistent with these findings, our results show that the dual H-bonding structure in our P_2_ configuration contributes to the enhanced stability of the TT conformer in aqueous solution (see table S2).

### Proton transfer and ring statistics

**Fig. 3.**
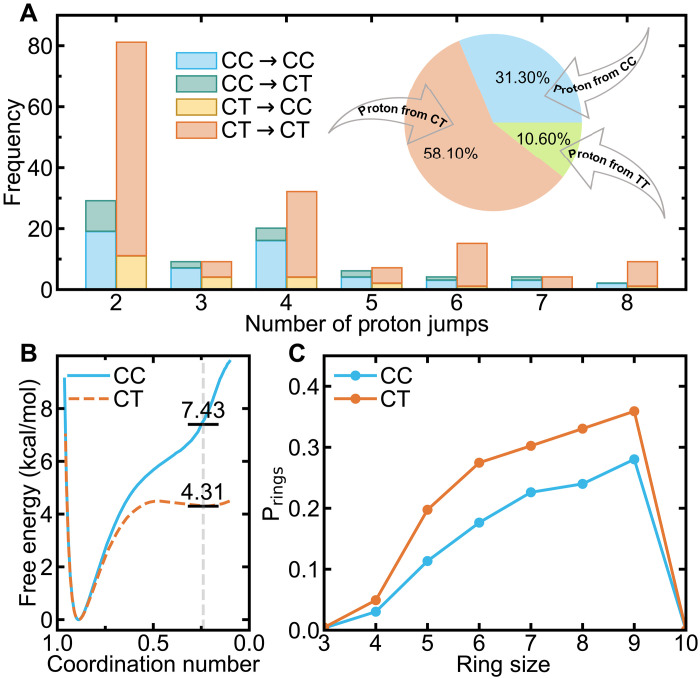
Proton transfer events of H_2_CO_3_ solutions. (**A**) Frequency analysis of the number of proton hops experienced by CC and CT conformers from dissociation to recombination, based on 300-ns DPMD simulations of 1 H_2_CO_3_ molecule solvated by 126 water molecules at 330 K; pie chart shows the fraction of proton sources in solution. (**B**) Dissociation free energy profiles along the hydrogen coordination number around the deprotonated oxygen atom for CC and CT conformers. The black horizontal line marks the dissociation free energy of the deprotonated acid. (**C**) Distribution of ring size that CC (blue) and CT (orange) conformers participate in.

Our simulations revealed frequent spontaneous conformational transitions and dissociations in aqueous H_2_CO_3_. These conformational transitions were categorized into two distinct mechanisms: direct (CT ⇒ CC) and indirect (CT ⇒ HCO_3_^−^ ⇒ CC) pathways (see fig. S9). The indirect mechanism is predominant (accounting for 89.02%), leading to substantial proton production and prompting further investigation into the sources of protons within the system. Statistically analyzing revealed that 58.1% of the dissociated protons originate from the CT conformer, as shown in the pie chart inset of Fig. 3A. This highlights the CT conformer’s disproportionately high contribution to proton transfer, despite its much lower mole percent compared to the CC conformer. This finding raises a key question: What drives the CT conformer’s unexpected high proton-donating ability?

To understand why the CT conformer contributes more protons than the CC conformer, we performed a detailed frequency analysis of conformational transitions and proton transfer events during H_2_CO_3_ dissociation, as shown in Fig. 3A. The most frequent event observed is double proton hopping, referred to as short-range proton transfer, in which a proton dissociates from H_2_CO_3_ to the nearest water molecule and subsequently returns. In contrast, less frequent multiple proton hopping events (involving more than two hops) constitute long-range proton transfer, in which a proton dissociates from H_2_CO_3_ and then travels along the H-bonding network before eventually returning to the H_2_CO_3_ molecule. Notably, dissociation events for the CT conformer occurred at a substantially higher frequency than those for the CC conformer in both short- and long-range transfers.

The elevated frequency of short-range proton transfer in the CT conformer can be attributed to its lower dissociation free energy. By calculating the dissociation free energies for each conformer, as determined by monitoring the hydrogen coordination number around the deprotonated oxygen atom (details in the Supplementary Materials), we obtained values of 7.43 kcal/mol for the CC conformer and 4.31 kcal/mol for the CT conformer, as illustrated in Fig. 3B. This difference explains the greater tendency of the CT conformer to undergo short-range proton transfer events.

Long-range proton transfer is influenced by the topology of the H-bonding network, as protons travel along closed rings formed by H-bonds, a mechanism that has been used to explain proton transfer in pure water (*8*, *59*) and solution (*60*–*63*). To investigate long-range proton transfer in H_2_CO_3_ solution, we analyzed the underlying H-bonding network with focus on closed ring structures (details in the Supplementary Materials). As shown in Fig. 3C, the CT conformer is typically involved in a greater number of rings regardless of ring size, whereas the CC conformer participates in fewer rings. This difference suggests that the CT conformer has a higher number of potential proton transfer pathways and thus contributes more frequently to long-range proton transfer events. Furthermore, the total number of rings for the CT conformer is substantially higher than that of the CC conformer, providing a network-based explanation for why the CT conformer is more frequently engaged in short-range proton transfer events.

### Electronic structure analysis

**Fig. 4.**
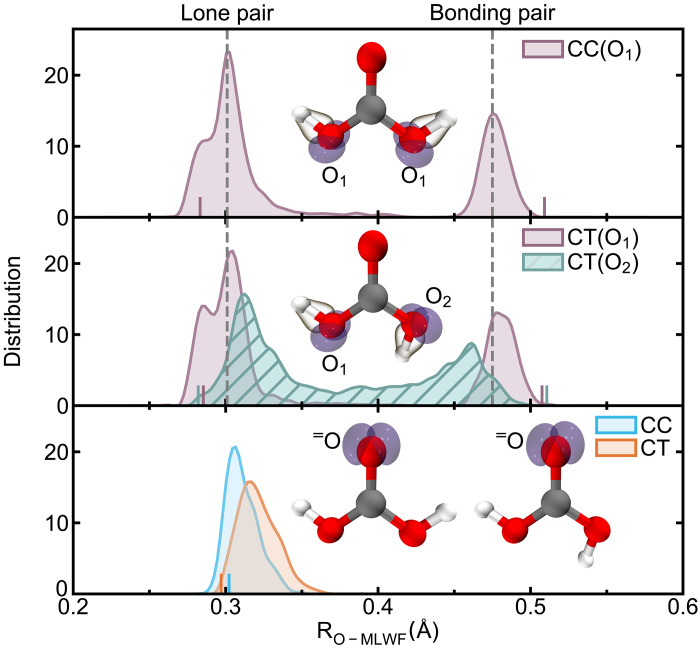
Electronic structure of CC and CT conformers in solution. Distributions of distances between intramolecular oxygen atoms in H_2_CO_3_ and the centers of the corresponding maximally localized Wannier functions (R_O-MLWF_ in Å) are shown. The top panel illustrates hydroxyl oxygens in the CC conformer, the middle panel shows the hydroxyl oxygens in the CT conformer, and the bottom panel represents the carbonyl oxygens in both CC and CT conformers. Short vertical lines denote the R_O-MLWF_ distances for gas phase H_2_CO_3_. Density isosurfaces for the MLWFs corresponding to lone- and bonding-pair electrons are depicted in purple and brown, respectively. Vertical dashed gray lines indicate the average distances from intramolecular oxygen atom to the Wannier centers of lone- and bonding-pairs of CC conformer.

The H-bonding topological network of the CT conformer markedly enhances its proton-donating ability, as discussed in the previous analysis. Here, we further show that the aqueous environment amplifies the symmetry breaking in the electronic structure of H_2_CO_3_, providing the quantum basis for increased deprotonation of the CT conformer. This finding is demonstrated by calculating and comparing the maximally localized Wannier function (MLWF) centers of H_2_CO_3_ in both gas and aqueous phases, as well as across different conformers (see Fig. 4). In the gas phase, the two hydroxyl oxygen atoms (O_1_) in the CC conformer exhibit identical electronic structures. In contrast, slight differences are observed between the two hydroxyl oxygen atoms (O_1_ and O_2_) in the CT conformer, due to their different orientations, reflecting the asymmetric influence of the carbonyl oxygen atom (^=^O). In aqueous solution, the hydrogen atoms of H_2_CO_3_ donate H-bonds, while the lone pairs on the oxygen atoms accept H-bonds from neighboring water molecules. The ability to donate or accept H-bonds, driven by electrostatic attraction, can be roughly estimated by the distance between the electron cloud and the nucleus (*10*). Although the R_O-MLWF_ distances of the CC and CT conformers are nearly identical in the gas phase, substantial differences emerge in the aqueous phase. In the CT conformer, both the lone and bonding pairs on O_2_ show increased hydrophilicity, while O_1_ remains largely unchanged with respect to the CC conformer. Structurally, the hydroxyl group of H_2_CO_3_ oriented away from the ^=^O cannot form intramolecular H-bonds with the carbonyl group, thereby becoming more exposed to interactions with the aqueous environment. In contrast, in the CC conformer, the ^=^O forms intramolecular H-bonds with both hydroxyl groups, reducing its sensitivity to the aqueous environment (see Fig. 4, bottom). This structural sensitivity is consistent with the insights gained from the electronic structure analysis.

### Proton transfer pathway

**Fig. 5.**
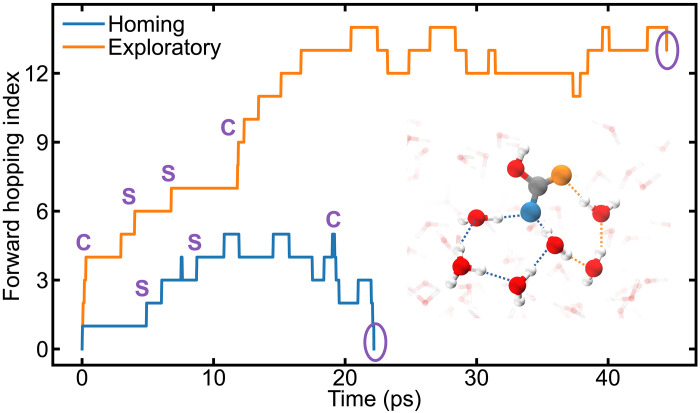
Proton transfer pathways in H_2_CO_3_ solution. Proton transfer pathways are categorized into homing and exploratory based on their end points. Regions labeled “S” represent single hopping events, while regions labeled “C” denote concerted hopping events. The inset illustrates two representative proton transfer pathways: the homing pathway (dashed blue line) and the exploratory pathway (dashed orange line). The blue atom indicates the starting point of the proton transfer.

Differences in electronic structures also influence the proton transfer pathways. To examine this, we selected two trajectory segments as examples of forward hopping and used the trajectory filtering method described in (*64*) to eliminate errors caused by rattling events during the simulations (details in the Supplementary Materials). As shown in Fig. 5, two distinct types of end points are observed at the completion of proton transfer: zero and nonzero. We classify these proton transfer pathways as homing and exploratory, respectively. The homing pathway corresponds to a dissociated proton returning to its original site (the deprotonated hydroxyl oxygen atom, shown in blue), while the exploratory pathway involves the proton binding to another site (the original carbonyl oxygen atom, shown in orange). We observe both single hopping and concerted hopping events in both the homing and exploratory pathways, with single hopping events being more dominant, consistent with findings in pure water (*64*). In particular, we find that CC and CT conformers both prefer homing pathways but with different degrees of preference. Although previous studies (*65*, *66*) have suggested that the negative charge in gas phase bicarbonate (HCO_3_^−^) is shared between the two uncoordinated oxygen atoms, we find that the two uncoordinated oxygens in HCO_3_^−^ exhibit substantial differences in the aqueous environment (fig. S12). The deprotonated hydroxyl oxygen atom in HCO_3_^−^, derived from the CC and CT conformers, shows stronger proton-capturing capability (hydrophilicity) than the original carbonyl oxygen atom. This contrast is more pronounced for HCO_3_^−^ derived from the CT conformer. Consequently, protons dissociated from the CT conformer show a stronger preference for the homing pathway, with 82.95% of protons selecting this pathway, compared to only 57.76% of protons from the CC conformer.

## DISCUSSION

In conclusion, our DPMD simulations provide an in-depth understanding of the dissociation dynamics of H_2_CO_3_ in aqueous solution on nanosecond timescales with ab initio accuracy, revealing diverse dissociation events, conformational transitions, and proton transfer behaviors. To begin with, we captured the spontaneous dissociation and conformation conversion of H_2_CO_3_, determining the relative stability of its conformers in the order CC > CT > TT, with the equilibrium mole percent of CC, CT, and TT conformers as 81.3, 12.4, and 0.3%, respectively. We also observed that the TT conformer forms a dual H-bonding structure with a single water molecule, aligning with experimental measurements of H_2_CO_3_-halide complexes. We find that although the CT conformer is less abundant, it serves as the dominant proton donor. The enhanced proton-donating ability of the CT conformer can be explained from two key perspectives. On the one hand, the CT conformer participates in a greater number of ring structures, which provides more pathways for proton transfer. Consistently, FES calculations show that the CT conformer has a lower dissociation free energy for initial proton transfer to the nearest water molecule compared to the CC conformer, further supporting its increased proton-donating propensity. On the other hand, the asymmetric influence of the carbonyl oxygen on the two hydroxyl groups, depending on their orientation, amplifies the symmetry breaking in the electronic structure. This increased asymmetry boosts the hydrophilicity of the CT conformer, contributing to its superior proton-donating ability. Last, our simulations reveal distinct proton transfer pathways, with both protons dissociated from the CC and CT conformers showing a preference for the homing pathway. However, this preference is stronger for protons dissociated from the CT conformer (82.95%) compared to the CC conformer (57.76%). This difference arises from the electronic structure of bicarbonate ions, where the disparity in proton capture capability between the deprotonated hydroxyl oxygen and the carbonyl oxygen is more pronounced in the CT-derived bicarbonate, favoring the homing pathway. These findings provide a comprehensive view of H_2_CO_3_ dissociation and proton transfer in solution, elucidating conformer-specific differences and emphasizing the unique role of the CT conformer in proton transfer. Given the role of H_2_CO_3_ in ocean acidification, pH regulation, and enzymatic catalysis, our findings provide atomic-level insights into its dissociation and proton transfer dynamics, advancing our understanding of fundamental processes in both environmental chemistry and biochemistry. Future studies exploring concentration effects may further enrich this understanding, particularly for more complex or concentrated systems.

## MATERIALS AND METHODS

### DP training

We used a concurrent learning scheme to generate the training set (*67*). Initially, we selected ~2400 snapshots from the BOMD simulations as the initial training set. For more accurate forces, we recalculated the energy and atomic forces of these configurations using the SCAN (*45*) functional with a kinetic energy cutoff of 150 rydberg (Ry). Then, an iterative active learning is performed, which consists of three steps: training, exploration, and labeling. In the training part, four separate DP models were independently trained using different random seeds based on the current training set. In the exploration part, we performed DPMD simulations using one of the four DP models. DPMD was initiated with the various configurations, including CC/CT/TT + 126 H_2_O, HCO_3_^−^ + H_3_O^+^ + 125 H_2_O, OH^−^ + H_3_O^+^ + 126 H_2_O, and 128 H_2_O. The configurations were simulated within a temperature range of 280 to 390 K and a pressure of 1 bar to ensure effective sampling. For each snapshot in the trajectories, all four models predicted the atomic forces, and we evaluated the accuracy of the models using the maximum SD (ζ). In the labeling step, we extracted snapshots with ζ values between 0.15 and 0.25 eV/Å. Subsequently, we calculated the energy and atomic forces of these snapshots using DFT and added them to the training set. We repeated this cycle of three steps until less than 1% of the configurations had ζ > 0.15 eV/Å. In addition, to complement the dataset, configurations involving protons dissociation were incorporated. These configurations were obtained through a combination of active learning and metadynamics simulations. In the end, our final training dataset comprises ~23,000 configurations.

The final training dataset was used to train our DP model using the DeePMD-kit package (*44*). We adopted the smooth version (*68*) of the DP model and used the full relative coordinates to construct the descriptor (“se_e2_a”). The cutoff radius of neighboring atoms is 6.0 Å. The size of the embedding network and fitting neural networks are set to be (25, 50, 100) and (240, 240, 240), respectively. The learning rate was set to decay from 1.0 × 10^−3^ to 1.0 × 10^−8^. The prefactors of the energy and the force in the loss function were adjusted from 0.02 to 1 and from 1000 to 1, respectively. The final model used for the production run was trained for a total of 1.6 × 10^7^ steps.

### Molecular dynamics simulations

For the BOMD simulations, we used the Quantum ESPRESSO (QE) software package (*69*, *70*) to perform BOMD simulations. The exchange-correlation energy is described using the SCAN functional because of its good performance in aqueous systems. Core electrons were modeled using norm-conserving Hamann-Schluter-Chiang-Vanderbilt pseudopotentials (*71*, *72*). Kohn-Sham wave functions were expanded with a plane-wave basis set with a kinetic energy cutoff of 85 Ry. The Brillouin zone was sampled at the gamma point. Simulations were performed in the NVT ensemble, with maintained temperature at 330 K via a Nosé-Hoover thermostat (*73*). The elevated temperature is to mimic the nuclear quantum effects on the liquid structure (*74*). The mass of hydrogen was scaled to that of deuterium. For the three conformers investigated (1 CC/CT/TT + 62 H_2_O), we used a periodic cubic cell of with 23.51 Bohr (12.44 Å) to simulate the water density of ~1 g/cm^3^. Each simulation was run for a duration of 65 ps, with a time step of 20 au (0.48 fs).

For the DPMD simulations, we used the trained DP model with the accuracy of ab initio SCAN functional to simulate the microscopic dynamics of aqueous H_2_CO_3_. The initial configurations consisted of 1 CC/CT/TT + 126 H_2_O. We performed three DPMD simulations for each conformer at 310, 330, and 350 K, each lasting 100 ns, using LAMMPS (*75*) along with the DeePMD-kit plugin. The simulations comprised 1 ns for equilibrium and the remaining time for analysis. In the simulations, we used the NPT ensemble, maintaining the temperature at 330 K and the pressure at 1 bar, using a Nosé-Hoover thermostat and barostat for temperature and pressure control, respectively. A time step of 0.5 fs was used, with the mass of hydrogen scaled to that of deuterium. To compare the RDFs with the BOMD, a 10-ns DPMD simulation was performed under the NVT ensemble using the same starting configuration as the BOMD.

### Free energy calculation

The free energy calculation corresponding to DP model validation is performed by using the well-tempered of metadynamics with adaptive Gaussians to perform the BOMD simulation. The simulation was performed using QE and a development version of PLUMED2 (*76*) supplemented by the Metadynamics module. The specific parameters for the BOMD simulation were the same as those used in the previous BOMD simulations, with the simulation duration set to 45 ps. For comparison, the DPMD simulation was performed for 10 ns using the same metadynamics framework and parameter settings. The two ^=^OC-O_OH_H dihedral angles α and β of H_2_CO_3_ were used as CVs in the construction of the via Metadynamics. The bias factor and initial Gaussian height for the 1 H_2_CO_3_ + 62 H_2_O system were set to be 10 and 0.5 kJ/mol, respectively. The Gaussian width was set to 0.1.

## Supplementary Materials

### This PDF file includes:

Supplementary Text Figs. S1 to S12 Tables S1 to S3 References
