# Supplementary material for: Ab initio deep neural network simulations reveal that carbonic acid dissociation is dominated by minority cis-trans conformers
Source: Sci Adv. 2025 May 7;11(19):eadu6525. doi: 10.1126/sciadv.adu6525 (PMC12057677; doi:10.1126/sciadv.adu6525)
Supplement: Supplementary file 1 — Supplementary Text Figs. S1 to S12 Tables S1 to S3 References [file sciadv.adu6525_sm.pdf]

Science Advances
AAAS

# Supplementary Materials for

## Ab initio deep neural network simulations reveal that carbonic acid dissociation is dominated by minority cis-trans conformers

Yueqi Zhao *et al.*

Corresponding author: Zhaoru Sun, sunzhr@shanghaitech.edu.cn

*Sci. Adv.* **11**, eadu6525 (2025)
DOI: 10.1126/sciadv.adu6525

**This PDF file includes:**

Supplementary Text
Figs. S1 to S12
Tables S1 to S3
References

## Supplementary Text

### DP Model Validation

We compared the energies and atomic forces as predicted by the DP model and SCAN DFT calculations to validate the DP model. The test set comprises approximately 404 configurations extracted from a 40 ns DPMD simulation of aqueous carbonic acid solution (1 $H_2CO_3$ + 126 $H_2O$) in the NPT ensemble at 330K and 1 bar. Parity plots of the atomic energy and forces, averaged over four independently trained DPs for both carbonic acid solutions, are shown in fig. S1. The DP model reproduces well the DFT energy and atomic forces. The root-mean-squared errors (MSE) of the energy and atomic force predicted by the DP model with respect to the DFT are 0.28 meV/atom and 0.057 eV/Å, respectively, indicating that the typical DPMD training accuracy has been achieved (*48, 50-52*).

To further validate the DP model, we conducted a more comprehensive validation of the three conformers of carbonic acid (CC, CT, TT). Figure S2 compares the radial distribution functions (RDFs) of water-water pairs and acid-water pairs obtained from BOMD and DPMD simulations. We can see a close agreement between the main features of the RDFs predicted by DPMD and BOMD. We found no notable differences between the RDFs of water-water pairs in aqueous solutions of the CC, CT, and TT conformers, so we present only the RDFs of water-water pairs in the aqueous solution of the CC conformer. Since the statistics for water-water pairs converge more easily than those for acid-water pairs, the DPMD-predicted results exhibit excellent agreement with the BOMD calculations. Figure S2 also shows that the presence of the acid does not perturb the structure of water, which retains its typical hydrogen-bonded structure (*46*). In addition to the acid-water RDFs presented in the main text, we also validated the C-$O_W$ and C-$H_W$ RDFs for the CC conformer in aqueous solutions. Similarly, for the CT and TT conformers, we validated the five types of acid-water RDFs mentioned above, and the DPMD-predicted results showed good agreement with those calculated by BOMD.

To provide a more rigorous validation of the DP model, we compared free energy calculations at the BOMD level with those from DPMD. Since reliable free energy surfaces (FES) at the BOMD level require bias potentials through enhanced sampling methods, we ensured consistency by using the same parameters in our DPMD validation to assess the relative stability of the three conformers. As shown in fig. S3, the depths of the valleys representing the three conformers are consistent between DPMD and BOMD, indicating that both methods yield similar results for the relative stability of the conformers. We also observed that the free energy barriers in BOMD are higher than those in DPMD, which can be attributed to the shorter BOMD simulation time, resulting in fewer barrier-crossing events and an overestimation of the free energy barriers. These results further confirm the accuracy and efficiency of our DP model.

### Acid Dissociation Constant

The acid dissociation constant ($K_a$) describes the deprotonation state of a molecule in a particular solvent. For convenience, the symbol $pK_a$ ($pK_a = -\log_{10} K_a$) is generally used to represent this value. We calculated the $pK_a$ of carbonic acid in aqueous solution by evaluating the free energy profile as a function of the proton coordination number to the solvent at 330K. These calculations were based on unbiased trajectories from the DPMD simulations. As shown in fig. S4, we estimate the $pK_a$ of the system by computing the free energy difference between the protonated and deprotonated states, yielding a $pK_a$ of 3.65. Our result agrees with experimental value of 3.53 (*13*), which shows that the DP model accurately captures the proton dissociation behavior of carbonic acid in solution.

Mole Percent

We performed DPMD simulations on a system consisting of 1 $H_2CO_3$ molecule solvated by 126 water molecules in the NPT ensemble at 330 K and 1 bar, with each simulation running for 300 ns. To assess the thermal stability of conformer populations within biologically and environmentally relevant temperature ranges, additional simulations were performed at 310 K and 350 K under identical conditions.

Table S1 summarizes the mole percent data for these conformers at 310 K, 330 K, and 350 K. The results indicate slight variations in mole percent with temperature, but these changes do not affect the relative stability of the CC and CT conformers. This supports our conclusion that the CC conformer is more abundant than the CT conformer over the temperature range studied.

Free Energy Surface

We mapped all configurations from the 300 ns unbiased DPMD trajectory onto the state space defined by the two $^{=}$OC-$O_{OH}$H dihedral angles $\alpha$ and $\beta$ of the $H_2CO_3$. Histogram statistics of the trajectory gave the probability density distribution $\rho\ (\alpha, \beta)$. The free energy distribution was then calculated using the following formula.

$$G(\alpha,\beta) = -k_B T ln\rho(\alpha,\beta)$$

Finally, we obtained the free energy surfaces for the three conformers of $H_2CO_3$, with the two dihedral angles α and β serving as the CVs.

Dissociation Free Energies

We explored the acid dissociation free energy profiles. As shown in fig. S10, we defined the distances $R_0$ and $R_1$ between the oxygens of the acid ($O_0$, $O_1$) and the two extra protons ($Ex_0$, $Ex_1$). For the dissociated state, we identified the nearest pair between the deprotonated oxygen atom of $HCO_3^-$ and the hydrogen atoms of $H_3O^+$ as $O_0$ and $Ex_0$, respectively. In the case of the Zundel proton ($H_5O_2^+$), we replace the hydrogen with oxygen as $Ex_0$. We then mapped $R_0$ and $R_1$ to the coordination number (CN) of the hydrogen atoms around the deprotonated oxygen atom using the following formula.

$$CN = \frac{1-\left(\frac{R}{r_c}\right)^m}{1-\left(\frac{R}{r_c}\right)^n}$$

The $r_c$ in the formula is the cutoff distance, $r_c$ = 1.3 Å. The exponential factors *m* and *n* have the values of 8 and 16, respectively. Small *CN* values, close to 0, mean that the atoms are weakly coordinated while larger values, close to 1, indicate strong coordination. Similar to the calculation of the free energy surface, the CV is changed from the dihedral angles (α, β) to the coordination number (CN), obtaining the dissociation free energy profiles according to the following formula.

$$G(CN) = -k_B T ln\rho(CN)$$

Finally, we obtained the dissociation free energy profiles for both the CC and CT conformers with the hydrogen coordination number around the deprotonated oxygen atom as the CV.

Ring Statistics

We adopted the method described in the article (*77*) and selected the R-β definition, where R represents the O-O distance and β represents the ∠HOO. We then define the PMF $W(R,\beta) = -kTlng(W,\beta)$ contours for which (from the MD simulation) are shown in fig. S11. For the hydrogen-bonds (H-bonds) between $H_2CO_3$ and water molecules, R=3 Å and β=25° are suitable choices, while for the H-bonds between water molecules, we still adopt the commonly used rectangular H-bonding cutoff values proposed by Luzar and Chandler (*78, 79*) (R=3.5 Å, β=30°).

We can gain more understanding of the H-bonding topology in aqueous carbonic acid solution by analyzing the ring statistics. The distribution of closed rings was not only used to study the topological networks in liquid water systems (*8, 80, 81*) but also in silicate structures (*82, 83*). We adopted the algorithm version of Gupta and Suzumura to search for closed rings and calculate the number of shortest closed-loop paths, each consisting of the oxygen atom in carbonic acid and two of its neighboring oxygen atoms in an undirected graph, where the vertices represent oxygen atoms and the edges represent hydrogen bonds. The specific definition of H-bonds is detailed in the Supplementary Discussion under the "Hydrogen Bonding Definition" section. We further calculated the distribution of rings involving carbonic acid using the formula $P_{rings}(i) = n_i / N$, where $n_i$ is the total number of *i*-membered rings in which a specific conformer of carbonic acid participates, and $N$ is the total number of snapshots analyzed for the corresponding conformer. For *i*-membered rings, a higher $P_{rings}$ value corresponds to a higher average number of *i*-membered rings.

Electronic Structure Calculation

The amphiphilicity analysis of solvated carbonic acid and bicarbonate in water was conducted using the maximally localized Wannier functions (MLWF) method (*84*) implemented in the Wannier90 code (*85*). First, the electronic ground state was obtained using the QE package with the SCAN functional. The calculations were performed using a single gamma-point. The cut-off energies for the wave function and the charge density were set to 150 Ry and 600 Ry, respectively. The ground-state wave functions were then projected onto Wannier functions using the Wannier90 code, and the MLWFs were the calculated.

The frames used for the electronic structure calculations in fig. S12 were selected based on the following criteria: the distance between the hydronium ion and both proton binding sites of bicarbonate must be greater than 4 Å. This ensures that the hydronium ion do not directly influence the two proton binding sites.

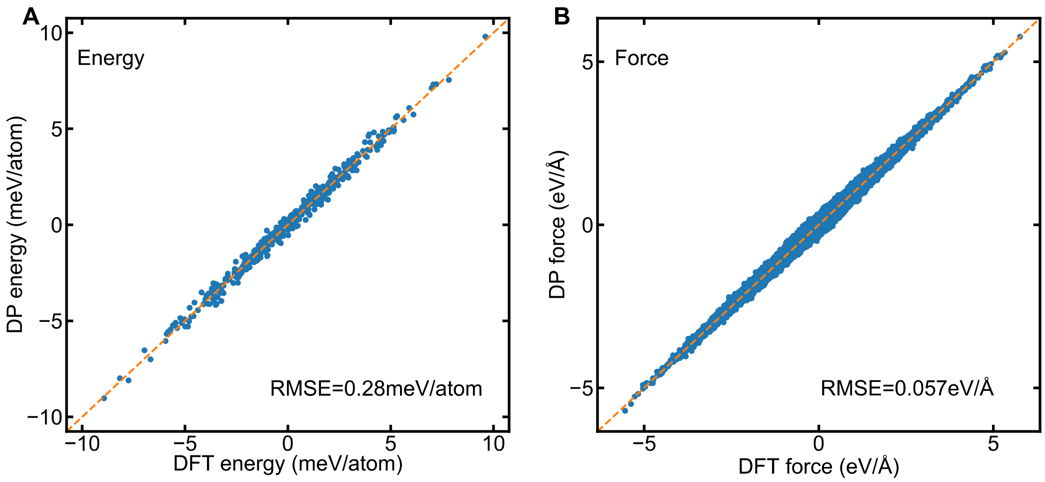


**Fig. S1.**

**Parity plot (DFT vs DP) of the energy (A) and atomic forces (B).** The energy values have been adjusted by shifting the average to zero to enhance visual clarity.

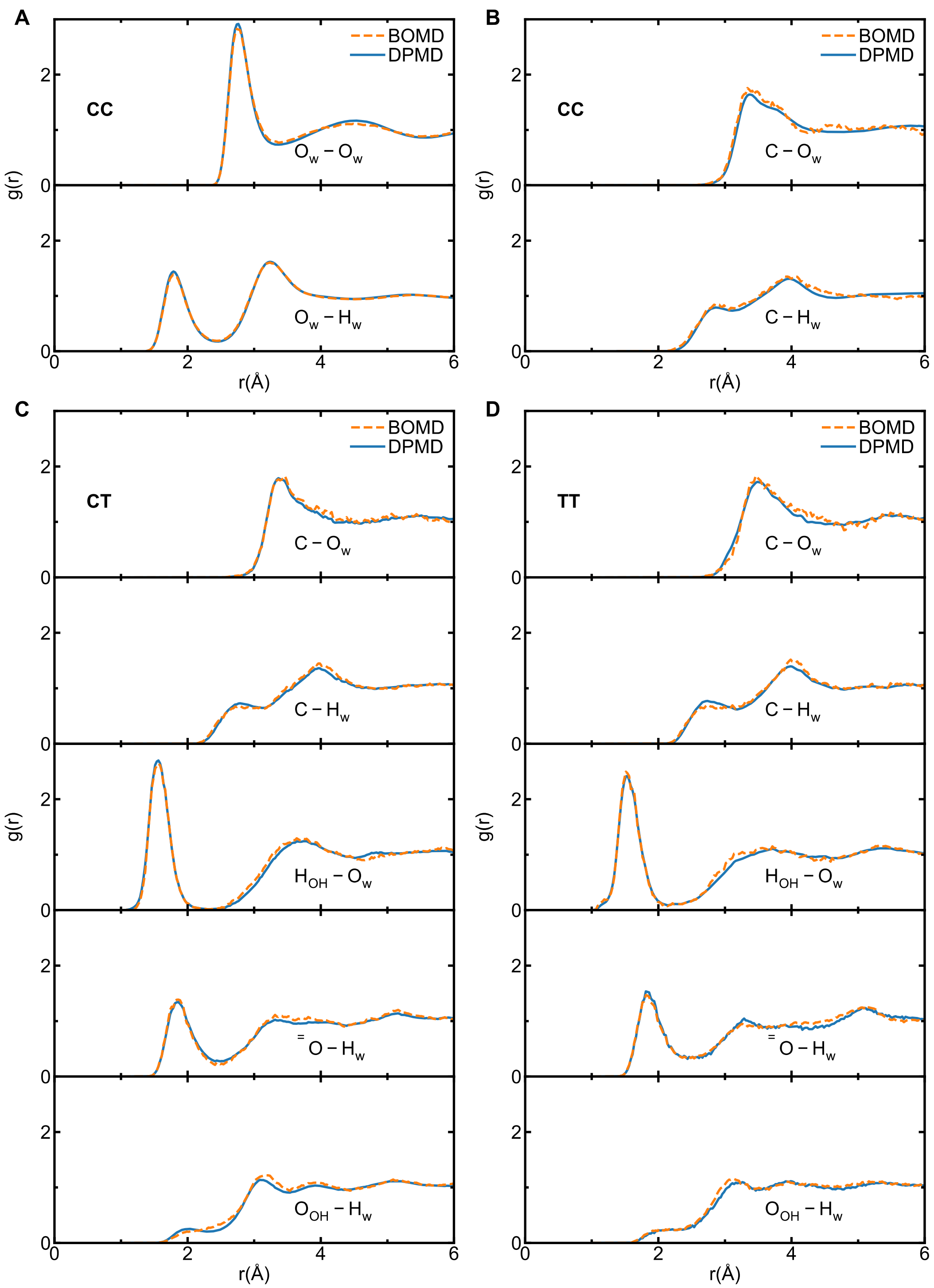


**Fig. S2.**

**Comparison of radial distribution functions (RDFs) obtained from BOMD and DPMD simulations:** (**A**) RDFs of water-water pairs in the CC conformer in aqueous solution, (**B**) RDFs of acid-water pairs in the CC conformer in aqueous solution, (**C**) RDFs of acid-water pairs in the CT conformer in aqueous solution, and (**D**) RDFs of acid-water pairs in the TT conformer in aqueous solution.

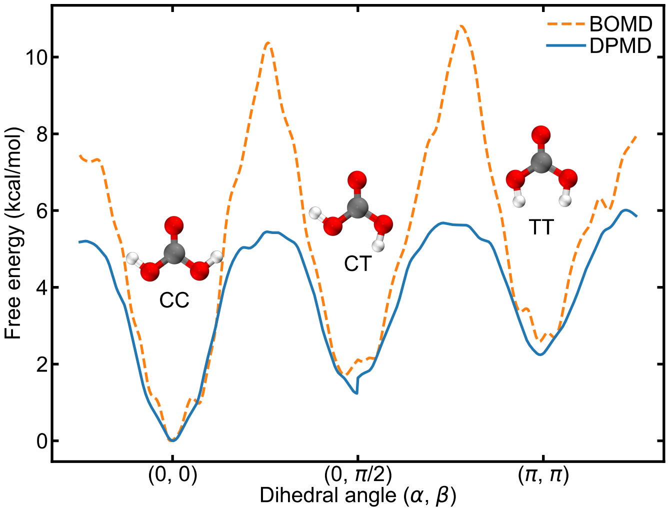


**Fig. S3.**

**Comparison of free energy profiles obtained from BOMD and DPMD metadynamics simulations.**

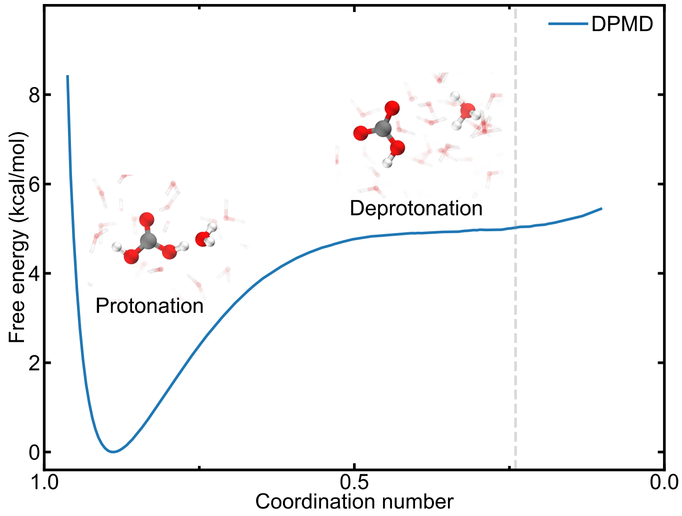


**Fig. S4.**

**Dissociation free energy profiles along the proton coordination number around the deprotonated oxygen atom for $H_2CO_3$ in aqueous solution.** The gray vertical dashed line represents the deprotonation state (coordination number = 0.24).

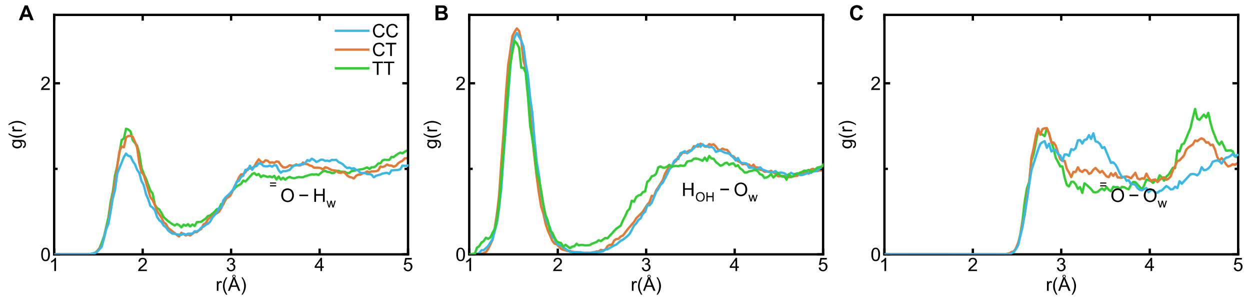


**Fig. S5.**

**The steric effect obtained from BOMD simulations.** (**A**) RDFs of $^{=}$O-$H_W$ pairs in aqueous $H_2CO_3$. (**B**) RDFs of $O_{OH}$-$O_W$ pairs in aqueous $H_2CO_3$. (**C**) RDFs of $^{=}$O-$O_W$ pairs in aqueous $H_2CO_3$.

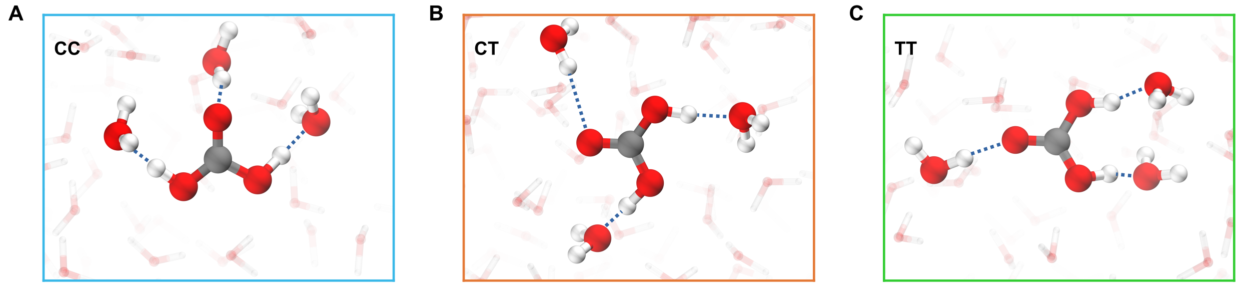


**Fig. S6.**
**The observed steric hindrance effects of CC (A), CT (B), and TT (C) conformer.**

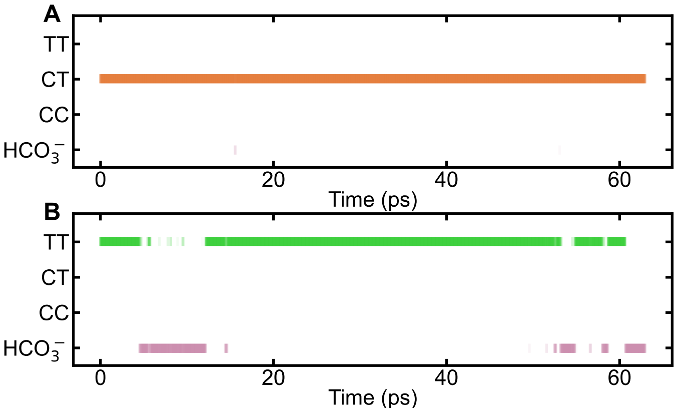


**Fig. S7.**

**Time evolution of carbon species in BOMD simulations:** (**A**) trajectory starting from the solution containing the CT conformer, and (**B**) trajectory starting from the solution containing the TT conformer.

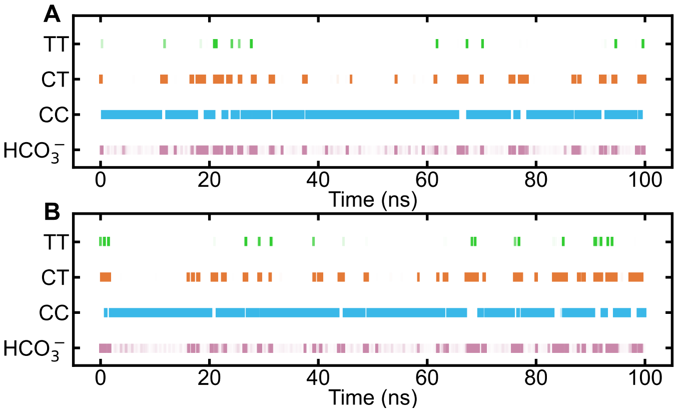


**Fig. S8.**

**Time evolution of carbon species in DPMD simulations:** (**A**) trajectory starting from the solution containing the CT conformer, and (**B**) trajectory starting from the solution containing the TT conformer.

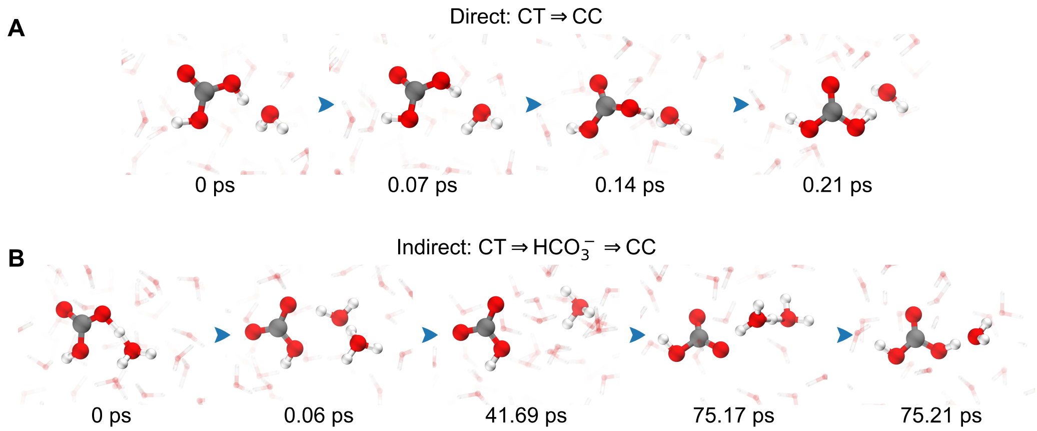


**Fig. S9.**

**Direct and indirect mechanisms of conformational changes in $H_2CO_3$.** (**A**) The representative direct conformational change pathway: CT ⇒ CC. (**B**) The representative indirect conformational change pathway: CT ⇒ $HCO_3^-$ ⇒ CC.

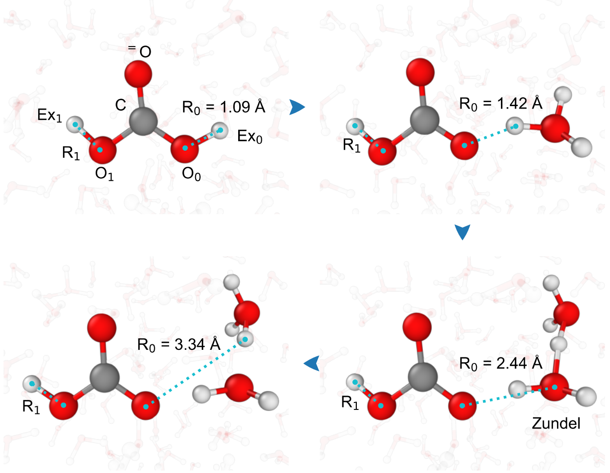


**Fig. S10.**

Represent snapshots of the acid dissociation and the definition of reaction coordinates.

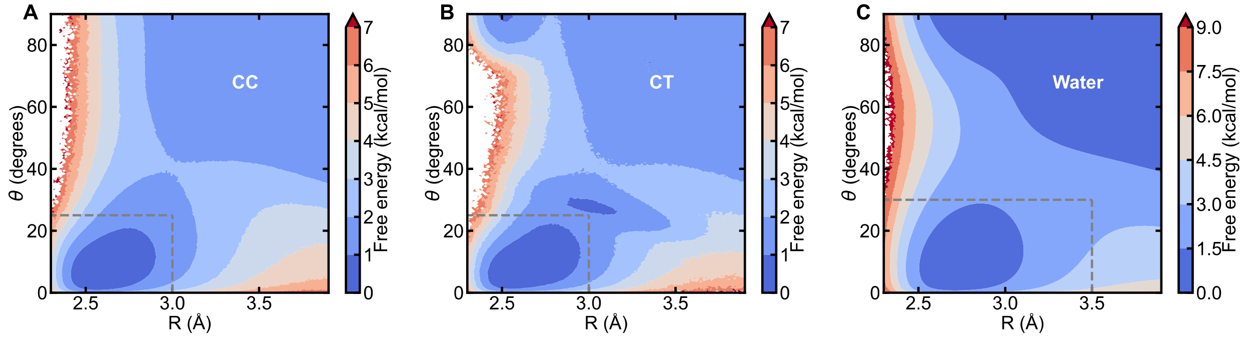


**Fig. S11.**

**Potential of PMF contour plots for (A) CC conformer, (B) CT conformer, and (C) pure water.** Dashed regions indicate suitable areas for defining hydrogen bonds.

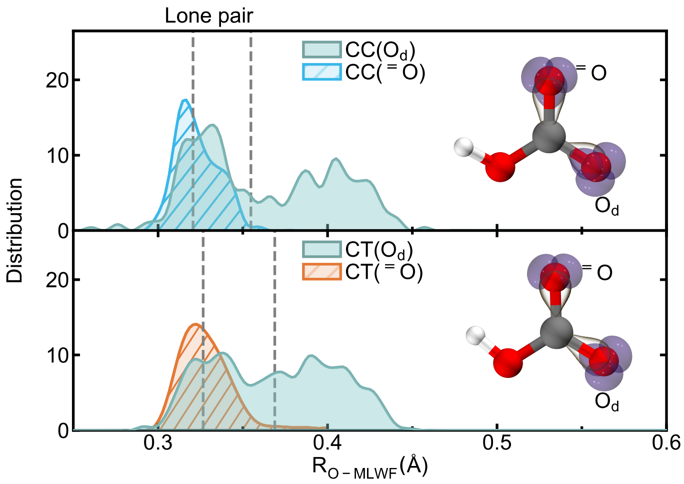


**Fig. S12.**

**Electronic structure of $HCO_3^-$ ions dissociated from $H_2CO_3$ in solution.** Distributions of distances between intramolecular oxygen atoms in $HCO_3^-$ and the centers of the corresponding maximally localized Wannier functions ($R_{O\text{-}MLWF}$ in Å). Different $HCO_3^-$ ions are shown: $HCO_3^-$ dissociated from the CC conformer (upper panel) and $HCO_3^-$ dissociated from the CT conformer (lower panel). Density isosurfaces of the MLWF for lone and bonding pair electrons are depicted in purple and brown, respectively. The vertical dashed gray lines represent the average distances from the intramolecular oxygen atoms to the Wannier centers of the lone pair electrons, for both the deprotonated hydroxyl atom ($O_d$) and carbonyl oxygen atom ($^=O$).

**Table S1.**

**Mole percent (%) of the three conformers in $H_2CO_3$ aqueous solution at 310K, 330K, and 350K.**

| Temperature | CC | CT | TT |
|---|---|---|---|
| 310K | 83.09% | 12.65% | 0.17% |
| 330K | 81.31% | 12.44% | 0.30% |
| 350K | 77.39% | 14.83% | 0.55% |

**Table S2.**

**Relative free energies (units: kcal/mol) of the three conformers in the gas phase** (*25*) **and aqueous solution, with the cis-cis (CC) conformer as the reference state.** The calculations highlight the contribution of the dual H-bonding structure to the enhanced stability of the trans-trans (TT) conformer in aqueous solution.

| Conformer | Gas phase | Aqueous phase |
|---|---|---|
| CC | 0.0 | 0.0 |
| CT | 1.5 | 1.8 |
| TT | 9.0 | 4.3 |

**Table S3.**

**Percentage of protons dissociated from the cis-cis (CC) and cis-trans (CT) conformers following the homing and exploratory pathways in aqueous solution.** The data show that the CT conformer has a stronger preference for the homing pathway compared to the CC conformer.

| Conformer | Homing pathway | Exploratory pathway |
|---|---|---|
| CC | 57.76% | 42.24% |
| CT | 82.95% | 17.05% |

## REFERENCES AND NOTES

1. N. Agmon, H. J. Bakker, R. K. Campen, R. H. Henchman, P. Pohl, S. Roke, M. Thämer, A. Hassanali, Protons and hydroxide ions in aqueous systems. *Chem. Rev.* **116**, 7642–7672 (2016).

2. A. W. Sakti, Y. Nishimura, H. Nakai, Recent advances in quantum-mechanical molecular dynamics simulations of proton transfer mechanism in various water-based environments. *WIREs Comput. Mol. Sci.* **10**, e1419 (2020).

3. H. Tai, S. Hirota, S. T. Stripp, Proton transfer mechanisms in bimetallic hydrogenases. *Acc. Chem. Res.* **54**, 232–241 (2021).

4. H. N. Raum, K. Modig, M. Akke, U. Weininger, Proton transfer kinetics in histidine side chains determined by pH-dependent multi-nuclear NMR relaxation. *J. Am. Chem. Soc.* **146**, 22284–22294 (2024).

5. K. S. Westendorff, M. J. Hülsey, T. S. Wesley, Y. Román-Leshkov, Y. Surendranath, Electrically driven proton transfer promotes Brønsted acid catalysis by orders of magnitude. *Science* **383**, 757–763 (2024).

6. L. Wang, J. Yan, Y. Hong, Z. Yu, J. Chen, J. Zheng, Ultrahigh-rate and ultralong-life aqueous batteries enabled by special pair-dancing proton transfer. *Sci. Adv.* **9**, eadf4589 (2023).

7. A. Hassanali, M. K. Prakash, H. Eshet, M. Parrinello, On the recombination of hydronium and hydroxide ions in water. *Proc. Natl. Acad. Sci. U.S.A.* **108**, 20410–20415 (2011).

8. A. Hassanali, F. Giberti, J. Cuny, T. D. Kühne, M. Parrinello, Proton transfer through the water gossamer. *Proc. Natl. Acad. Sci. U.S.A.* **110**, 13723–13728 (2013).

9. C. T. Wolke, J. A. Fournier, L. C. Dzugan, M. R. Fagiani, T. T. Odbadrakh, H. Knorke, K. D. Jordan, A. B. McCoy, K. R. Asmis, M. A. Johnson, Spectroscopic snapshots of the proton-transfer mechanism in water. *Science* **354**, 1131–1135 (2016).

10. M. Chen, L. Zheng, B. Santra, H.-Y. Ko, R. A. DiStasio Jr., M. L. Klein, R. Car, X. Wu, Hydroxide diffuses slower than hydronium in water because its solvated structure inhibits correlated proton transfer. *Nat. Chem.* **10**, 413–419 (2018).

11. A. Bogot, M. Poline, M. Ji, A. Dochain, A. Simonsson, S. Rosén, H. Zettergren, H. T. Schmidt, R. D. Thomas, D. Strasser, The mutual neutralization of hydronium and hydroxide. *Science* **383**, 285–289 (2024).

12. A. Gomez, W. H. Thompson, D. Laage, Neural-network-based molecular dynamics simulations reveal that proton transport in water is doubly gated by sequential hydrogen-bond exchange. *Nat. Chem.* **16**, 1838–1844 (2024).

13. D. Aminov, D. Pines, P. M. Kiefer, S. Daschakraborty, J. T. Hynes, E. Pines, Intact carbonic acid is a viable protonating agent for biological bases. *Proc. Natl. Acad. Sci. U.S.A.* **116**, 20837–20843 (2019).

14. D. Pines, J. Ditkovich, T. Mukra, Y. Miller, P. M. Kiefer, S. Daschakraborty, J. T. Hynes, E. Pines, How acidic is carbonic acid? *J. Phys. Chem. B* **120**, 2440–2451 (2016).

15. A. Stirling, I. Pápai, $H_2CO_3$ forms via $HCO_3^-$ in water. *J. Phys. Chem. B* **114**, 16854–16859 (2010).

16. S. Jovanovic, P. Jakes, S. Merz, D. T. Daniel, R.-A. Eichel, J. Granwehr, *In operando* NMR investigations of the aqueous electrolyte chemistry during electrolytic $CO_2$ reduction. *Commun. Chem.* **6**, 268 (2023).

17. K. Adamczyk, M. Prémont-Schwarz, D. Pines, E. Pines, E. T. J. Nibbering, Real-time observation of carbonic acid formation in aqueous solution. *Science* **326**, 1690–1694 (2009).

18. N. Stolte, D. Pan, Large presence of carbonic acid in $CO_2$-rich aqueous fluids under Earth's mantle conditions. *J. Phys. Chem. Lett.* **10**, 5135–5141 (2019).

19. D. Pan, G. Galli, A first principles method to determine speciation of carbonates in supercritical water. *Nat. Commun.* **11**, 421 (2020).

20. R. Dettori, D. Donadio, Carbon dioxide, bicarbonate and carbonate ions in aqueous solutions under deep Earth conditions. *Phys. Chem. Chem. Phys.* **22**, 10717–10725 (2020).

21. N. Stolte, J. Yu, Z. Chen, D. A. Sverjensky, D. Pan, Water–gas shift reaction produces formate at extreme pressures and temperatures in deep Earth fluids. *J. Phys. Chem. Lett.* **12**, 4292–4298 (2021).

22. W. Hage, K. R. Liedl, A. Hallbrucker, E. Mayer, Carbonic acid in the gas phase and its astrophysical relevance. *Science* **279**, 1332–1335 (1998).

23. T. Loerting, C. Tautermann, R. T. Kroemer, I. Kohl, A. Hallbrucker, E. Mayer, K. R. Liedl, On the surprising kinetic stability of carbonic acid ($H_2CO_3$). *Angew. Chem. Int. Ed. Engl.* **39**, 891–894 (2000).

24. J. Bernard, R. G. Huber, K. R. Liedl, H. Grothe, T. Loerting, Matrix isolation studies of carbonic acid—The vapor phase above the β-polymorph. *J. Am. Chem. Soc.* **135**, 7732–7737 (2013).

25. P. P. Kumar, A. G. Kalinichev, R. J. Kirkpatrick, Dissociation of carbonic acid: Gas phase energetics and mechanism from *ab initio* metadynamics simulations. *J. Chem. Phys.* **126**, 204315 (2007).

26. H. P. Reisenauer, J. P. Wagner, P. R. Schreiner, Gas-phase preparation of carbonic acid and its monomethyl ester. *Angew. Chem. Int. Ed. Engl.* **53**, 11766–11771 (2014).

27. T. Mori, K. Suma, Y. Sumiyoshi, Y. Endo, Spectroscopic detection of isolated carbonic acid. *J. Chem. Phys.* **130**, 204308 (2009).

28. T. Mori, K. Suma, Y. Sumiyoshi, Y. Endo, Spectroscopic detection of the most stable carbonic acid, cis-cis $H_2CO_3$. *J. Chem. Phys.* **134**, 044319 (2011).

29. T. Loerting, J. Bernard, Aqueous carbonic acid ($H_2CO_3$). *ChemPhysChem* **11**, 2305–2309 (2010).

30. D. Laage, J. T. Hynes, A molecular jump mechanism of water reorientation. *Science* **311**, 832–835 (2006).

31. O. Marsalek, T. E. Markland, Quantum dynamics and spectroscopy of ab initio liquid water: The interplay of nuclear and electronic quantum effects. *J. Phys. Chem. Lett.* **8**, 1545–1551 (2017).

32. Y. Yun, R. Z. Khaliullin, Y. Jung, Correlated local fluctuations in the hydrogen bond network of liquid water. *J. Am. Chem. Soc.* **144**, 13127–13136 (2022).

33. S. Daschakraborty, P. M. Kiefer, Y. Miller, Y. Motro, D. Pines, E. Pines, J. T. Hynes, Reaction mechanism for direct proton transfer from carbonic acid to a strong base in aqueous solution I: Acid and base coordinate and charge dynamics. *J. Phys. Chem. B* **120**, 2271–2280 (2016).

34. S. Daschakraborty, P. M. Kiefer, Y. Miller, Y. Motro, D. Pines, E. Pines, J. T. Hynes, Reaction mechanism for direct proton transfer from carbonic acid to a strong base in aqueous solution II: Solvent coordinate-dependent reaction path. *J. Phys. Chem. B* **120**, 2281–2290 (2016).

35. M. de la Puente, R. David, A. Gomez, D. Laage, Acids at the edge: Why nitric and formic acid dissociations at air-water interfaces depend on depth and on interface specific area. *J. Am. Chem. Soc.* **144**, 10524–10529 (2022).

36. G. Bussi, A. Laio, Using metadynamics to explore complex free-energy landscapes. *Nat Rev Phys* **2**, 200–212 (2020).

37. E. Grifoni, G. Piccini, M. Parrinello, Microscopic description of acid–base equilibrium. *Proc. Natl. Acad. Sci. U.S.A.* **116**, 4054–4057 (2019).

38. X. Liu, X. Lu, R. Wang, H. Zhou, In silico calculation of acidity constants of carbonic acid conformers. *J. Phys. Chem. A* **114**, 12914–12917 (2010).

39. D. Polino, E. Grifoni, R. Rousseau, M. Parrinello, V.-A. Glezakou, How collective phenomena impact $CO_2$ reactivity and speciation in different media. *J. Phys. Chem. A* **124**, 3963–3975 (2020).

40. M. Invernizzi, M. Parrinello, Rethinking metadynamics: From bias potentials to probability distributions. *J. Phys. Chem. Lett.* **11**, 2731–2736 (2020).

41. A. S. Kamenik, S. M. Linker, S. Riniker, Enhanced sampling without borders: On global biasing functions and how to reweight them. *Phys. Chem. Chem. Phys.* **24**, 1225–1236 (2022).

42. C. S. Tautermann, A. F. Voegele, T. Loerting, I. Kohl, A. Hallbrucker, E. Mayer, K. R. Liedl, Towards the experimental decomposition rate of carbonic acid ($H_2CO_3$) in aqueous solution. *Chemistry* **8**, 66–73 (2002).

43. A. D. Kulkarni, Molecular hydration of carbonic acid: Ab initio quantum chemical and density functional theory investigation. *J. Phys. Chem. A* **123**, 5504–5516 (2019).

44. L. Zhang, J. Han, H. Wang, R. Car, W. E, Deep potential molecular dynamics: A scalable model with the accuracy of quantum mechanics. *Phys. Rev. Lett.* **120**, 143001 (2018).

45. J. Sun, A. Ruzsinszky, J. P. Perdew, Strongly constrained and appropriately normed semilocal density functional. *Phys. Rev. Lett.* **115**, 036402 (2015).

46. M. Chen, H.-Y. Ko, R. C. Remsing, M. F. Calegari Andrade, B. Santra, Z. Sun, A. Selloni, R. Car, M. L. Klein, J. P. Perdew, X. Wu, Ab initio theory and modeling of water. *Proc. Natl. Acad. Sci. U.S.A.* **114**, 10846–10851 (2017).

47. R. Wang, V. Carnevale, M. L. Klein, E. Borguet, First-principles calculation of water p$K_a$ using the newly developed SCAN functional. *J. Phys. Chem. Lett.* **11**, 54–59 (2020).

48. C. Zhang, S. Yue, A. Z. Panagiotopoulos, M. L. Klein, X. Wu, Dissolving salt is not equivalent to applying a pressure on water. *Nat. Commun.* **13**, 822 (2022).

49. J. Guo, L. Zhou, A. Zen, A. Michaelides, X. Wu, E. Wang, L. Xu, J. Chen, Hydration of $NH_4^+$ in water: bifurcated hydrogen bonding structures and fast rotational dynamics. *Phys. Rev. Lett.* **125**, 106001 (2020).

50. T. Wen, L. Zhang, H. Wang, E. Weinan, D. J. Srolovitz, Deep potentials for materials science. *Mater. Futures* **1**, 022601 (2022).

51. A. S. Raman, A. Selloni, Modeling the solvation and acidity of carboxylic acids using an *ab initio* deep neural network potential. *J. Phys. Chem. A* **126**, 7283–7290 (2022).

52. B. Wen, M. F. Calegari Andrade, L.-M. Liu, A. Selloni, Water dissociation at the water–rutile $TiO_2$ (110) interface from ab initio-based deep neural network simulations. *Proc. Natl. Acad. Sci. U.S.A.* **120**, e2212250120 (2023).

53. P. P. Kumar, A. G. Kalinichev, R. J. Kirkpatrick, Hydrogen-bonding structure and dynamics of aqueous carbonate species from Car−Parrinello molecular dynamics simulations. *J. Phys. Chem. B* **113**, 794–802 (2009).

54. S. K. Reddy, S. Balasubramanian, Carbonic acid: Molecule, crystal and aqueous solution. *Chem. Commun.* **50**, 503–514 (2014).

55. M. Galib, G. Hanna, Mechanistic insights into the dissociation and decomposition of carbonic acid in water via the hydroxide route: An ab initio metadynamics study. *J. Phys. Chem. B* **115**, 15024–15035 (2011).

56. C. A. Wight, A. I. Boldyrev, Potential energy surface and vibrational frequencies of carbonic acid. *J. Phys. Chem.* **99**, 12125–12130 (1995).

57. D. A. Thomas, E. Mucha, M. Lettow, G. Meijer, M. Rossi, G. Von Helden, Characterization of a trans-trans carbonic acid-fluoride complex by infrared action spectroscopy in helium nanodroplets. *J. Am. Chem. Soc.* **141**, 5815–5823 (2019).

58. H. Zhang, W. Cao, Q. Yuan, L. Wang, X. Zhou, S. Liu, X.-B. Wang, Spectroscopic evidence for intact carbonic acid stabilized by halide anions in the gas phase. *Phys. Chem. Chem. Phys.* **22**, 19459–19467 (2020).

59. S. Yan, B. Wang, H. Lin, Tracking the delocalized proton in concerted proton transfer in bulk water. *J. Chem. Theory Comput.* **19**, 448–459 (2023).

60. E. Méndez, P. E. Videla, D. Laria, Collective proton transfers in cyclic water–Ammonia tetramers: A path integral machine-learning study. *J. Phys. Chem. A* **127**, 1839–1848 (2023).

61. J. Cuny, A. A. Hassanali, Ab initio molecular dynamics study of the mechanism of proton recombination with a weak base. *J. Phys. Chem. B* **118**, 13903–13912 (2014).

62. K. De Wispelaere, B. Ensing, A. Ghysels, E. J. Meijer, V. Van Speybroeck, Complex reaction environments and competing reaction mechanisms in zeolite catalysis: Insights from advanced molecular dynamics. *Chem. A Eur. J.* **21**, 9385–9396 (2015).

63. G. Murdachaew, G. M. Nathanson, R. B. Gerber, L. Halonen, Deprotonation of formic acid in collisions with a liquid water surface studied by molecular dynamics and metadynamics simulations. *Phys. Chem. Chem. Phys.* **18**, 29756–29770 (2016).

64. C. Arntsen, C. Chen, P. B. Calio, C. Li, G. A. Voth, The hopping mechanism of the hydrated excess proton and its contribution to proton diffusion in water. *J. Chem. Phys.* **154**, 194506 (2021).

65. E. Garand, T. Wende, D. J. Goebbert, R. Bergmann, G. Meijer, D. M. Neumark, K. R. Asmis, Infrared spectroscopy of hydrated bicarbonate anion clusters: $HCO_3^-(H_2O)_{1-10}$. *J. Am. Chem. Soc.* **132**, 849–856 (2010).

66. B. Denegri, M. Matić, O. Kronja, The role of negative hyperconjugation in decomposition of bicarbonate and organic carbonate anions. *ChemistrySelect* **1**, 5250–5259 (2016).

67. Y. Zhang, H. Wang, W. Chen, J. Zeng, L. Zhang, H. Wang, W. E, DP-GEN: A concurrent learning platform for the generation of reliable deep learning based potential energy models. *Comput. Phys. Commun.* **253**, 107206 (2020).

68. L. Zhang, J. Han, H. Wang, W. Saidi, R. Car, W. E, "End-to-end symmetry preserving inter-atomic potential energy model for finite and extended systems" in *Advances in Neural Information Processing Systems*, S. Bengio, H. Wallach, H. Larochelle, K. Grauman, N. Cesa-Bianchi, R. Garnett, Eds. (Curran Associates Inc., 2018) vol. 31; https://proceedings.neurips.cc/paper_files/paper/2018/file/e2ad76f2326fbc6b56a45a56c59fafdb-Paper.pdf.

69. P. Giannozzi, O. Andreussi, T. Brumme, O. Bunau, M. B. Nardelli, M. Calandra, R. Car, C. Cavazzoni, D. Ceresoli, M. Cococcioni, N. Colonna, I. Carnimeo, A. D. Corso, S. de

Gironcoli, P. Delugas, R. A. Di Stasio Jr., A. Ferretti, A. Floris, G. Fratesi, G. Fugallo, R. Gebauer, U. Gerstmann, F. Giustino, T. Gorni, J. Jia, M. Kawamura, H.-Y. Ko, A. Kokalj, E. Küçükbenli, M. Lazzeri, M. Marsili, N. Marzari, F. Mauri, N. L. Nguyen, H.-V. Nguyen, A. Otero-de-la-Roza, L. Paulatto, S. Poncé, D. Rocca, R. Sabatini, B. Santra, M. Schlipf, A. P. Seitsonen, A. Smogunov, I. Timrov, T. Thonhauser, P. Umari, N. Vast, X. Wu, S. Baroni, Advanced capabilities for materials modelling with QUANTUM ESPRESSO. *J. Phys. Condens. Matter* **29**, 465901 (2017).

70. P. Giannozzi, S. Baroni, N. Bonini, M. Calandra, R. Car, C. Cavazzoni, D. Ceresoli, G. L. Chiarotti, M. Cococcioni, I. Dabo, A. Dal Corso, S. De Gironcoli, S. Fabris, G. Fratesi, R. Gebauer, U. Gerstmann, C. Gougoussis, A. Kokalj, M. Lazzeri, L. Martin-Samos, N. Marzari, F. Mauri, R. Mazzarello, S. Paolini, A. Pasquarello, L. Paulatto, C. Sbraccia, S. Scandolo, G. Sclauzero, A. P. Seitsonen, A. Smogunov, P. Umari, R. M. Wentzcovitch, QUANTUM ESPRESSO: A modular and open-source software project for quantum simulations of materials. *J. Phys. Condens. Matter* **21**, 395502 (2009).

71. D. Vanderbilt, Optimally smooth norm-conserving pseudopotentials. *Phys. Rev. B* **32**, 8412–8415 (1985).

72. D. R. Hamann, M. Schlüter, C. Chiang, Norm-conserving pseudopotentials. *Phys. Rev. Lett.* **43**, 1494–1497 (1979).

73. G. J. Martyna, M. L. Klein, M. Tuckerman, Nosé–Hoover chains: The canonical ensemble via continuous dynamics. *J. Chem. Phys.* **97**, 2635–2643 (1992).

74. J. A. Morrone, R. Car, Nuclear quantum effects in water. *Phys. Rev. Lett.* **101**, 017801 (2008).

75. A. P. Thompson, H. M. Aktulga, R. Berger, D. S. Bolintineanu, W. M. Brown, P. S. Crozier, P. J. I. Veld, A. Kohlmeyer, S. G. Moore, T. D. Nguyen, R. Shan, M. J. Stevens, J. Tranchida, C. Trott, S. J. Plimpton, LAMMPS - A flexible simulation tool for particle-based materials modeling at the atomic, meso, and continuum scales. *Comput. Phys. Commun.* **271**, 108171 (2022).

76. G. A. Tribello, M. Bonomi, D. Branduardi, C. Camilloni, G. Bussi, PLUMED 2: New feathers for an old bird. *Comput. Phys. Commun.* **185**, 604–613 (2014).

77. R. Kumar, J. R. Schmidt, J. L. Skinner, Hydrogen bonding definitions and dynamics in liquid water. *J. Chem. Phys.* **126**, 204107 (2007).

78. A. Luzar, D. Chandler, Hydrogen-bond kinetics in liquid water. *Nature* **379**, 55–57 (1996).

79. A. Luzar, D. Chandler, Effect of environment on hydrogen bond dynamics in liquid water. *Phys. Rev. Lett.* **76**, 928–931 (1996).

80. A. C. Belch, S. A. Rice, The distribution of rings of hydrogen-bonded molecules in a model of liquid water. *J. Chem. Phys.* **86**, 5676–5682 (1987).

81. M. Matsumoto, A. Baba, I. Ohmine, Topological building blocks of hydrogen bond network in water. *J. Chem. Phys.* **127**, 134504 (2007).

82. S. V. King, Ring configurations in a random network model of vitreous silica. *Nature* **213**, 1112–1113 (1967).

83. X. Yuan, A. N. Cormack, Efficient algorithm for primitive ring statistics in topological networks. *Comput. Mater. Sci.* **24**, 343–360 (2002).

84. N. Marzari, D. Vanderbilt, Maximally localized generalized Wannier functions for composite energy bands. *Phys. Rev. B* **56**, 12847–12865 (1997).

85. G. Pizzi, V. Vitale, R. Arita, S. Blügel, F. Freimuth, G. Géranton, M. Gibertini, D. Gresch, C. Johnson, T. Koretsune, J. Ibañez-Azpiroz, H. Lee, J.-M. Lihm, D. Marchand, A. Marrazzo, Y. Mokrousov, J. I. Mustafa, Y. Nohara, Y. Nomura, L. Paulatto, S. Poncé, T. Ponweiser, J. Qiao, F. Thöle, S. S. Tsirkin, M. Wierzbowska, N. Marzari, D. Vanderbilt, I. Souza, A. A. Mostofi, J. R. Yates, Wannier90 as a community code: New features and applications. *J. Phys. Condens. Matter* **32**, 165902 (2020).
